# Mental health and adjustment to juvenile idiopathic arthritis: Level of agreement between parent and adolescent reports according to Strengths and Difficulties Questionnaire and Adolescent Outcomes Questionnaire

**DOI:** 10.1371/journal.pone.0173768

**Published:** 2017-03-10

**Authors:** Ewa Misterska, Dominika Kaminiarczyk-Pyzałka, Karolina Adamczak, Katarzyna A. Adamczyk, Marek Niedziela, Maciej Głowacki, Jakub Głowacki

**Affiliations:** 1 Department of Pedagogy and Psychology, University of Security, Poznan, Poland; 2 Department of Paediatric Endocrinology and Rheumatology, 2nd Chair of Paediatrics, Poznan University of Medical Sciences, Poznan, Poland; 3 Institute of Psychology, Adam Mickiewicz University, Poznan, Poland; 4 Department of Pediatric Orthopaedics and Traumatology, Poznan University of Medical Sciences, Poland; 5 HCP Medical Centre, Poznan, Poland; IRCCS E. Medea, ITALY

## Abstract

The aims of this study were threefold. Firstly, to analyze the psychometric properties of the Polish-language Pediatric Outcomes Data Collection Instrument (PODCI) questionnaire in the self-report Adolescent Outcomes Questionnaire (adolescents, 11–18 years of age) and in the parent-report Adolescent Outcomes Questionnaire (completed by a parent or guardian of an adolescent aged 11–18 years). Secondly, to determine the level of agreement between parents and adolescents in rating dysfunction in juvenile idiopathic arthritis (JIA) and thirdly, to examine associations between psychological adjustments of patients to JIA and disease as well as their socio-demographic characteristics. The study sample consisted of 52 participants. 26 adolescents between the ages of 11 and 18 years with a diagnosis of JIA and 26 parents were considered for inclusion. Disease course was classified as pauciarticular (n = 12, 46.2%) and polyarticular (n = 14, 53.8%). Participants completed the PODCI (self- and parent- report) twice and the Strengths and Difficulties Questionnaire-25 (SDQ-25). Considering the distribution of results regarding PODCI normative scores, 73.1% of parents and 69.2% of patients scored below 50 on the Global Functioning Scale; that is lower than the average for the general healthy population. Regarding the parent report, the total score of the SDQ-25 equaled 11.86 (SD 2.66), whereas the patient report equaled 11.23 (SD 2.78). The study groups do not differ significantly in regards to either the PODCI or the SDQ-25 results. Parents and adolescents with JIA appear to hold very similar perceptions of patients' health. Greater differences emerge as disease severity and age of patients increase. Excellent internal consistency, intrarater and test-retest reliability of the Global Functioning Scale have been confirmed in the Polish version of the PODCI, the questionnaire may therefore aid identification of patients reporting significant problems in this group.

## Introduction

Information acquired from children and their parents is important in the assessment of childhood health-related quality of life (HRQL) [1]. Juvenile idiopathic arthritis (JIA) and the treatment of it present numerous challenges to adolescents with this disease and to their families. For example, treatment requirements and medical appointments, which may also be a financial burden, can restrict a family's activities [2–5].

Meta-analysis results reveal that children and adolescents with chronic arthritis are more likely to suffer psychological adjustment problems compared to the control group, according to the parent report. Adjustment problems and issues with internalizing symptoms affect children with arthritis in particular, this pattern is also supported by the findings of epidemiological research and literature reviews of psychological adjustment in pediatric chronic illness [6–9]. However, adolescents with arthritis were not deemed to face a higher risk of poor self-concept or self-esteem problems in comparison to the control group [6–9].

The aspect of how parents adjust to JIA has generally been overlooked, despite nearly two-thirds of parents declaring they experience moderate-to-severe difficulties in this area [10]. Viewed from a systems perspective, functioning of parents and families may be negatively affected should a childhood chronic disease such as JIA be diagnosed [11]. As well as having to provide care for a child with special needs and all the additional demands that such care entails, parents must cope with their own personal feelings of uncertainty and the sense that they are no longer in control [12].

Parents are involved in the process of adaptation to JIA and familial characteristics such as maternal adjustment, marital and familial adjustment, family support and cohesiveness affect how well the child adapts to JIA [13]. Furthermore, mild to moderate rates of problematic adjustment among parents of children with a chronic illness can be seen [14]. In particular, mothers are at high risk of problematic adjustment since they are more likely to be traditionally responsible for the daily care of the child and provision of his or her medical needs [15].

According to Duffy et al. [16], children aged 9 years or over with JIA and spondyloarthritis recorded good agreement in relation to their parents concerning the level of dysfunction across a wide range of general symptoms including physical and psychosocial function. This implies that information acquired from children with chronic arthritis and their parents on the extent to which the disease affects the child's quality of life is reliable and can be of value in such assessments. One area where a difference was noted regarded anxiety, depression and behavior, based on the Strengths and Difficulties Questionnaire-25 (SDQ-25). The children's self-report in this field did not reveal a higher risk of maladjustment whereas parental reports did. Further research is necessary to clarify how significant this discrepancy is, although it is suspected that parents are more aware of their child's emotional state [17].

The Pediatric Outcomes Data Collection Instrument (PODCI) is a questionnaire-based tool used in the assessment of physical function in children with chronic health disorders. It has been applied in studies of childhood arthritis despite not being initially designed to be used in cases of pediatric rheumatic diseases [18]. The purpose of the PODCI is to assess patient functioning in terms of overall health, pain, ability to participate in normal daily activities and other types of more vigorous activities that are usually associated with young people. The target group is patients with general health problems, specifically those related to bone and muscle conditions. First published in 1994, it was updated in 2005 [19,20].

Another method widely applied to assess behavioral and emotional problems as well as the relationships of children and adolescents is the SDQ-25. Each area of the SDQ-25 contains five items making a total of 25 questions. As a research instrument, the SDQ-25 is brief and covers key aspects of common childhood and adolescent psychopathology, it includes strengths as well as difficulties and therefore parents—particularly across the general population—find it more acceptable [21,22]. For these reasons the SDQ-25 is a tool valued by researchers and clinicians. It is available in a version for parents, a second version for teachers—both of which cover children and adolescents, and a third self-reported version which is used with adolescents only.

As the PODCI had never before been applied to evaluate the efficacy of treatment in JIA patients, it was first necessary to analyze the psychometric properties of the Polish-language PODCI questionnaire in the self-report Adolescent Outcomes Questionnaire (adolescents aged 11–18 years) and in the parent-report Adolescent Outcomes Questionnaire (completed by a parent or guardian of an adolescent aged 11–18 years) in relation to the SDQ-25. Secondly, a further aim of this study was to determine discrepancies between the reports submitted by parents and those by adolescents with JIA of physical and psychological function in terms of the SDQ-25 and PODCI. Thirdly, we aimed to examine associations between psychological adjustments of patients to JIA and disease as well as their socio-demographic characteristics. We hypothesized that if we adapt PODCI questionnaires to Polish cultural conditions, we will achieve tools that are equivalent to the original English-language methods, in terms of internal consistency, intrarater and test-retest reliability, concurrent and construct validity and responsiveness. Moreover, taking into account the results of previous research, e.g. the Duffy et al study [16], it was assumed that some discrepancies between parental reports and those by adolescents with JIA of physical and psychological function might occur, especially in regard to rating the patient’s emotional state.

## Material and methods

### Study design

The study design was cross-sectional and consecutively recruited participants—patients with JIA (aged 11–18 years) and their parents—for study inclusion from one academic clinic during routine visits. This age range was chosen, as the instruments used were already standardized for ages of 11–18 yrs.

### Selection to the study

The target participants for the presented study were adolescents aged 11 to 18 years with a diagnosis of juvenile idiopathic arthritis for at least 3 months, with one or more joints affected when diagnosis was established, together with their parents.

Exclusion criteria were as follows: evidence of comorbid cognitive deficits (e.g., mental retardation) or other comorbid chronic illness. Recruitment of patients and parents was carried out in the clinic. The questionnaires were completed either on site or at the patient's home, in which case the study packets were returned via postage-paid mail.

### Procedures

The study received approval from the Poznan University of Medical Sciences Bioethics Committee. During routine clinic visits, patients who met the entry criteria and their parents, were approached by a research assistant who explained that the purpose of the study was to gather information about the experience of having a rheumatic disease. Written informed consent was obtained from adolescents and parents included in the study.

The study participants completed the questionnaires at the same time but in isolation to make certain that parents and patients did not consult each other about their responses. The researcher was on hand to answer any queries from the participants regarding the content of the questionnaires.

All the participants completed the study measures twice with a two-day interval in accordance with the requirements of the intrarater reliability study of the Polish version of PODCI.

### Study sample

There were 52 participants in this study sample: 26 were adolescents with a diagnosis of JIA and 26 were parents. Among the 26 adolescent patients the number of joints affected at the time of inclusion into the study was as follows: no joints affected by arthritis: 19.23% (n = 5), one joint affected: 38.46% (n = 10), two joints affected: 15.38% of patients (n = 4), four joints affected: 7.69% of patients (n = 2), five joints affected: 3.85% of patients (n = 1), six joints affected: 7.69% of patients (n = 2), eight joints affected: 3.85% of patients (n = 1) and twelve joints affected by arthritis: 3.85% of patients (n = 1). Pauciarticular disease course was determined in 12 patients (46.2%), polyarticular in 14 patients (53.8%). Table 1 shows the detailed characteristics, including demographics, of the participating adolescents.

**Table 1:** Study sample characteristics.

Study sample characteristics	Mean (SD), range	No. (%)
Gender (M/F) (patients)		14 (53.8%)/12(46.2%)
Age of patients (yrs)	15.04 (2.01), 11–18	
Weight (kg)	48.23 (21.73), 17–72	
Height (cm)	153.23 (15.76), 122–173	
Disease course		
Pauciarticular		12 (46.2%)
Polyarticular		14 (53.8%)
Disease duration (months)	11.62 (21.73), 3–108	
Pharmacotherapy		
Methotrexate * (mg/ m2 of body surface per week)	15.32 (3.40), 10–20.0	23 (88.46%)
Glucocorticoids (mg/kg/per day)	5.56 (3.47), 2.0–16.0	18 (69.23%)
Cyclosporine A (mg/kg/per day)	103.13 (33.9), 50.0–150.0	8(30.78%)
Etanercept mg/kg/dose 2 times per week	36.13 (11.89), 25.0–50.0	8(30.78%)

* Methotrexate was taken orally (p.o).

The sample comprised 14 (53.8%) boys and 12 (46.2%) girls, all aged between 11 to 18 years (M = 15.04, SD = 2.01), and their parents. Of the 26 patients, thirteen patients (50%) lived in a city.

### Study measures

The PODCI questionnaire in a version of the Adolescent Outcomes Questionnaire (self-report for adolescents aged 11–18 years) contained 83 items and was completed twice by the JIA patients. Likewise, parents completed the PODCI in a version of the Adolescent Outcomes Questionnaire (parent-report to be completed by a parent or guardian of an adolescent, aged 11–18 years) which contained 86 items, twice [18]. There are five subscales within the PODCI: upper extremity and physical function, transfer and mobility tasks, sports/physical functioning, pain/comfort and happiness. The Global Functioning scale regards the mean of the “mean of items” values for the first 4 of these subscales. The majority of the items apply a categorical scale with 3–6 choices; in the remainder, respondents are requested to circle “yes” to all the responses which apply to the patient [18]. A scoring worksheet in Microsoft Excel 2003 where raw scores for all scales can be recorded is available from the AAOS website. The raw scores entered for each scale are converted to a standard score based on the mean of items within that particular scale. All the items in a scale are recalibrated to the same metric, ranging in value from 0–5 for each item. The scores for all the items in a scale are then averaged over the number of items answered. The next step is to multiply the mean of the rescaled values by a constant so that each scale has a final range of values between 0–100.

In the Excel worksheets standardized scores (from 0–100) are calculated such that as the scores increase, disability levels decrease, representing better functioning. For purposes of comparison across the scales, data from the general United States (US) population was transformed for each scale setting a normative score of 50 and SD of 10. Therefore, a patient who scores over 50 on a particular scale is rated above the average for the general healthy population, and a scale score of below 50 indicates the patient is below this average. The PODCI possesses appropriate psychometric properties and can be applied in clinical research. Only Korean and Spanish versions of the PODCI have been made available to date [18,23,24].

Patients and parents were asked to assess patients' perceptions of their mental health across the 5 subscales of the Polish versions of the SDQ-25 self-report and parent-report [21]. Goodman SDQ-25’ five areas of clinical interest are covered: emotional problems, hyperactivity, peer problems, conduct problems and pro-social behavior [25].

The scope of possible answers in the SDQ-25 ranges from “Not True”, to “Somewhat True” and “Certainly True”. Answers receive a score of 0–2 points where, depending on the template, “Not True” may score either 0 or 2 points [21,25]. The sum of the points from each separate item within a subscale provides the score—from 0 to 10—for each subscale. The score from four of the subscales (hyperactivity, emotional problems, conduct and relationship) are combined to generate a total score of difficulties from 0–40. The pro-social scale points are not taken into account in the total score of difficulties, as the absence of pro-social behavior is considered to be conceptually different from the presence of psychological difficulties. Cut-off points, available online at www.sdqinfo.com, were used to define scores according to the following categories: “normal” or “psychiatric disorder: unlikely”, “borderline” or “psychiatric disorder: possible” and “abnormal” or “psychiatric disorder: probable” [21,25].

### Adaptation procedure of the Polish version of the Adolescent Outcomes Questionnaire: Self- and parent- report for youth 11–18 years of age

The Polish cultural adaptation of the PODCI followed the recommendations suggested by Beaton et al. in 2000 [26]. First, two native Polish translators (working alone) translated the English version of the PODCI into Polish. Next, the project authors and both translators compared and synthesized the translations. Then, two native bilingual English speakers, back-translated the Polish version of PODCI into English. The fourth stage involved a meeting between the all four translators, an orthopedic surgeon, and a psychologist during which all the translations were reviewed and verified. Any inconsistencies in the translations which arose were checked and corrected and the result was a pre-final version of the PODCI.

### Statistical analysis

Means, minimal and maximal values, standard deviations and 95% confidence intervals were identified for quantitative features. The number of units for specific categories of a given characteristic and their relative percentage values was provided for the quality field. The Shapiro-Wilk test was used to verify the normality of data distribution. The Spearman's rank correlation coefficient (rS) was applied to determine the associations between quantitative characteristics. The Mann-Whitney test was used to examine the mean level differences in the domain of quantitative characteristics in regard to gender and disease course. Wilcoxon signed ranks tests were used in the comparison of patient and parent perceptions of patient functioning.

Regarding PODCI (Polish version) psychometric properties, internal consistency was evaluated using Cronbach's alpha. An alpha value of 0.85 was considered acceptable [27,28]. Floor (% of patients with the minimal score) and ceiling (% of patients with the maximum score) effects were also analyzed. An excellent result rating is when no floor or ceiling effects are identified, < 20% = adequate and > 20% = poor. Intrarater reliability relates to the stability of data recorded by one individual across two trials, and the Intraclass Correlation Coefficient (ICC) was applied to evaluate this aspect [27]. The results were set as follows: ICC > 0.75 = excellent reliability, 0.40 to < 0.74 = adequate, < 0.40 = poor reliability. Test-retest reliability was assessed using the Pearson correlation coefficient allowing immediate comparison with other studies which had also applied this technique. The accepted Pearson correlations were follows: excellent = r ≥ 0.75, adequate = 0.40–0.74, poor = ≤ 0.40 [27,28]. In the case of the PODCI, concurrent validity—the extent to which scores from a new measure are related to scores from a measure administered at the same time—was assessed by correlation with scores from an accepted criterion measure, here we used Spearman’s rank correlations with the SDQ-25 scores. The Spearman’s correlation coefficient was employed to control construct validity evaluated by associations with active joint count.

Responsiveness was determined as the effect size [29]. To calculate effect sizes, the mean absolute change score was divided by the standard deviation of the baseline score. The interpretation of the magnitude of the effect size was based on Cohen’s rule-of-thumb: 0.2–0.5 is considered a small effect size, 0.5–0.8 = moderate effect, 0.8 or greater = large effect [29]. p = 0.05 marked the borderline value of statistical significance. Any test results with a value higher than p = 0.05 were treated as statistically irrelevant. Statistical analysis was performed using the Statistica program.

## Results

### Descriptive statistics

Table 2 presents the PODCI subscales and the Global Functioning Scale results expressed as mean standardized and normative scores, SD, range and 95% CI. The mean standardized Global Functioning Scale for parent reports equaled 82.09 (SD 13.61) and for adolescent reports 83.14 (13.93). The mean normative Global Functioning Scale score for parent and adolescent reports was 32.80 (18.44) and 34.23 (18.88), respectively, below the general healthy population average. The highest scores representing less disability and better functioning emerged in the Upper Extremity and Physical Function and Transfer and Basic Mobility Scales, in both the parent and adolescent reports (detailed results within particular subscales are presented in Table 2).

**Table 2:** Distribution of standardized and normative scores within the PODCI Adolescent Outcomes Questionnaire (parent- and self- report for youth 11–18 years of age).

PODCI Adolescent Outcomes Questionnaire: Standardized scores	Mean (SD) (parent-report/self-report)	Min-Max (parent-report/self-report)	95% Confidence Intervals (from-to) (parent-report/self-report)
Upper Extremity and Physical Function Scale	95.49 (7.45)/96.79 (7.48)	75-100/70.83–100	92.48–98.50/93.77–99.82
Transfer and Basic Mobility Scale	96.40 (5.15)/96.88 (5.21)	82.58-100/81.81–100	94.319–98.48/94.78–98.99
Sports/Physical Functioning Scale	73.19 (19.74)/74.58 (20.92)	34.72-100/32.64–100	65.22–81.16/66.13–83.03
Pain/Comfort Scale	63.27 (29.14)/64.31 (27.96)	15-100/15-100	51.50–75.04/53.02–75.61
Happiness Scale	56.15 (20.85)/56.92 (19.24)	10-100/25-95	47.73–64.57/49.15–64.69
GLOBAL FUNCTIONING SCALE	82.09 (13.61)/83.14 (13.93)	59.40-100/52.27–100	76.59–87.58/77.52–88.77
PODCI Adolescent Outcomes Questionnaire: Normative scores*			
Upper Extremity and Physical Function Scale	43.54 (14.80)/46.13 (14.87)	2.82–52.50/-5.46–52.50	37.56–49.52/40.13–52.14
Transfer and Basic Mobility Scale	44.13 (11.10)/45.17 (11.23)	14.35–51.88/12.71–51.88	39.64–48.61/40.63–49.70
Sports/Physical Functioning Scale	31.27(18.06)/45.17 (19.14)	-3.93–55.79/-5.84–55.79	23.97–38.56/24.80–40.27
Pain/Comfort Scale	35.85 (17.00)/36.46 (16.26)	7.78–57.21/7.79–57.22	29.01–42.70/29.90–43.03
Happiness Scale	35.68 (11.71)/36.11 (10.81)	9.75–57.50/18.18–57.50	30.95–40.41/31.75–40.48
GLOBAL FUNCTIONING SCALE	32.80 (18.44)/34.23 (18.88)	2.05–57.08/1.88–57.08	25.35–40.25/26.61–41.85

*http://www.aaos.org/research/outcomes/outcomes_documentation.asp#norm

Regarding the distribution of the PODCI normative scores results, 26.9% of parents and 30.8% of patients achieved a score ≥ 50 on the Global Functioning Scale, a result that is within or above average in relation to the general healthy population. 73.1% of parents and 69.2% of patients scored under 50 on this scale, lower than the average for the general healthy population. Table 3 presents results within particular subscales.

**Table 3:** Distribution of normative scores within the PODCI Adolescent Outcomes Questionnaire (parent- and self-report for youth 11–18 years of age).

PODCI Adolescent Outcomes Questionnaire	% of patients scoring ≥ 50 parent-report/patient-report	% of patients scoring < 50 parent-report/patient-report
Upper Extremity and Physical Function Scale	65.4%/76.9%	34.6%/23.1%
Transfer and Basic Mobility Scale	53.8%/65.4%	46.2%/34.6%
Sports/Physical Functioning Scale	19.2%/23.1%	80.8%/76.9%
Pain/Comfort Scale	30.8%/34.6%	69.2%/65.4%
Happiness Scale	7.7%/3.8%	92.3%/96.2%
GLOBAL FUNCTIONING SCALE	26.9%/30.8%	73.1%/69.2%

A detailed analysis of the distribution of scores for the SDQ-25 parent version as well as the patient version can be seen in Tables 4 and 5. Individual domain results and total scores are presented. In the parent report, the SDQ-25 total score equaled 11.86 (SD 2.66), whereas it was 11.23 (SD 2.78) in the patient report.

**Table 4:** Distribution of scores within SDQ-25 according to parent and patient reports.

SDQ-25 subscales	Mean (SD)	95% CI	Range (Min-Max)	Mean (SD)	95% CI	Range (Min-Max)
	Parent report			Patient report		
Emotional symptoms	1.23 (1.24)	0.73–1.73	0–5	1.12 (1.24)	0.61–1.62	0–4
Conduct problems	1.96 (0.77)	1.65–2.27	1–4	1.92 (0.80)	1.60–2.24	0–3
Hyperactivity/inattention	4.00 (1.23)	3.50–4.50	2–7	3.69 (1.05)	3.27–4.12	2–6
Peer relation problems	4.65 (1.13)	4.10–5.11	2–7	4.50 (0.99)	4.10–4.90	3–7
Pro-social behavior	1.73 (1.73)	1.03–2.43	0–7	1.50 (1.48)	0.90–2.10	0–5
Total score	11.85 (2.66)	10.77–12.92	8–18	11.23 (2.78)	10.11–12.35	7–19

**Table 5:** Distribution of “normal”, “borderline” and “abnormal” SDQ-25 scores: Patients and parents.

SDQ-25 subscales*	Parent report			Patient report		
	A (%)	B (%)	C (%)	A (%)	B (%)	C (%)
Emotional symptoms (patient/parent)	96,2	0	3,8	100	0	0
Conduct problems (patient/parent)	80,8	15,4	3,8	100	0	0
Hyperactivity/inattention (patient/parent)	88,5	7,7	3,8	96,2	3,8	0
Peer relation problems (patient/parent)	88.5	7,7	3.8	15.4	73,1	11.5
Pro-social behavior (patient/parent)	92,3	3,8	3,8	96,2	3,8	0
Total score (patient/parent)	80,8	7,7	11,5	92,3	7,7	0

*Note*. *A* psychiatric disorder: unlikely; *B* psychiatric disorder: possible; *C* psychiatric disorder: probable;

*patients/parents;

SDQ-25-The Strengths and Difficulties Questionnaire-25

After application of the accepted cut-off points [24–26], the overall results acquired from all SDQ-25 domain and general results fell into the “psychiatric disorder: unlikely” band in both the patient and parent versions. In the patient questionnaires, the following percentages of respondents with low scores (falling within the normal range) were recorded: emotional symptoms subscale—100.00%, conduct problems– 100.00%, hyperactivity—96.20%, interpersonal relationships—11.40%, prosocial behavior—96.20%, total score—92.30%. The parent questionnaires rating patient mental health delivered the following percentages of children with low scores (falling within normal): emotional symptoms subscale—96.2%, conduct problems 80.80%, hyperactivity—88.50%, interpersonal relationships—88.50%, prosocial behavior– 92.30%, total score—80.80% (a detailed distribution of “borderline” and “abnormal” SDQ-25 patient scores is presented in Table 5).

### The Polish version of the PODCI Adolescent Outcomes Questionnaire: Parent- and self-report for youth 11–18 years of age: Psychometric properties

PODCI internal consistency: parent- and self- report for adolescents 11–18 years of age within particular subscales and the Global Functioning Scale, floor and ceiling effects, intrarater reliability (ICC values), test-retest reliability and responsiveness, are displayed in Table 6.

**Table 6:** Internal consistency, floor and ceiling effects, intrarater reliability, test-retest reliability and responsiveness of the PODCI Adolescent Outcomes Questionnaire (parent-and self-report for youth 11–18 years of age).

PODCI Adolescent Outcomes Questionnaire (parent report/patient report)	Internal consistency (Cronbach’s alpha)	Floor effect	Ceiling effect	Intrarater reliability (ICC)	Test-retest reliability (Pearson correlation coefficient)	Responsiveness (Effect size)
Upper Extremity and Physical Function Scale	0.63/0.85*	0%/0%	65%/77%	1.0/1.0	0.99/1.0	0.043/0.043
Transfer and Basic Mobility Scale	0.73/0.76	0%/0%	54%/65%	1.0/1.0	0.80/0.98	0.181/0.089
Sports/Physical Functioning Scale	0.90/0.92	0%/0%	4%/12%	1.0/1.0	0.97/1.0	0.133/0.081
Pain/Comfort Scale	-	0%/0%	27%/23%	1.0/1.0	0.95/0.99	0.131/0.090
Happiness Scale	0.88/0.82	0%/0%	0%/0%	1.0/1.0	0.95/0.94	0.194/0.230
GLOBAL FUNCTIONING SCALE	0.88/0.89	0%/0%	4%/12%	1.0/1.0	0.66/0.99	0.127/0.078

*parent-report/self-report

The Global Functioning Scale Cronbach’s alpha values were excellent and equaled 0.88 and 0.89 in the parent- and self- report, respectively. No floor effects regarding the Global Functioning Scale were recorded, although the ceiling effects for this Scale reached 4% and 12% for parent- and self- report, respectively. These results are considered excellent and adequate, respectively.

For responsiveness, the effect sizes in the Global Functioning Scale were small, we recorded 0.127 in the parent-report and 0.078 in the self-report.

Temporal stability (test-retest reliability) results based on the Pearson correlation coefficient, were excellent at 0.95 (parent-report) and 0.94 (self-report). Intrarater reliability (ICC) equaled 1.00 and 1.00 for parent- and self-report, respectively. Table 6 presents detailed results within particular subscales. To verify concurrent validity, we evaluated correlations between the PODCI Adolescent Outcomes Questionnaire: parent- and self-report for adolescents aged 11–18 years and the SDQ-25 according to parent and patient reports.

Regarding associations between parent-reports, a statistically significant correlation was recorded between the PODCI Global functioning scale and the Total score of the SDQ-25 (rs = -0.43). This was also detected between all PODCI and SDQ-25 subscales (for details see Table 7).

**Table 7:** Correlation between PODCI Adolescent Outcomes Questionnaire (parent- and self-report for youth 11–18 years of age) and SDQ-25 according to parent and patient.

PODCI Adolescent Outcomes Questionnaire: parent report/ SDQ-25: parent report	Emotional symptoms	Conduct problems	Hyperactivity/inattention	Peer relation problems	Pro-social behavior	Total score
Upper Extremity and Physical Function Scale	rs = -0.64*	rs = -0.21	rs = -0.15	rs = -0.26	rs = -0.13	rs = -0.54*
Transfer and Basic Mobility Scale	rs = -0.32	rs = -0.18	rs = -0.43*	rs = -0.20	rs = 0.09	rs = -0.50*
Sports/Physical Functioning Scale	rs = -0.43*	rs = -0.05	rs = -0.35	rs = -0.17	rs = -0.05	rs = -0.47*
Pain/Comfort Scale	rs = -0.37	rs = 0.18	rs = -0.33	rs = -0.19	rs = 0.09	rs = -0.30
Happiness Scale	rs = -0.19	rs = -0.01	rs = -0.12	rs = -0.05	rs = 0.05	rs = -0.25
GLOBAL FUNCTIONING SCALE	rs = -0.47*	rs = 0.04	rs = -0.37	rs = -0.19	rs = 0.01	rs = -0.43*
PODCI Adolescent Outcomes Questionnaire: patient report/ SDQ-25: patient report	Emotional symptoms	Conduct problems	Hyperactivity/inattention	Peer relation problems	Pro-social behavior	Total score
Upper Extremity and Physical Function Scale	rs = -0.30	rs = -0.41*	rs = 0.17	rs = -0.33	rs = -0.33	rs = 0.24
Transfer and Basic Mobility Scale	rs = -0.08	rs = -0.55*	rs = -0.14	rs = -0.17	rs = -0.17	rs = 0.32
Sports/Physical Functioning Scale	rs = -0.16	rs = -0.39	rs = -0.02	rs = -0.21	rs = -0.21	rs = 0.04
Pain/Comfort Scale	rs = -0.18	rs = -0.50*	rs = -0.11	rs = -0.10	rs = -0.10	rs = 0.07
Happiness Scale	rs = -0.10	rs = -0.56*	rs = -0.02	rs = -0.27	rs = -0.27	rs = 0.07
GLOBAL FUNCTIONING SCALE	rs = -0.19	rs = -0.46*	rs = -0.08	rs = -0.16	rs = -0.16	rs = 0.10

* p < 0.05

In regard to the patient reports, no statistically significant correlations between the PODCI and SDQ-25 results were noted (see Table 7).

### Associations between patient-parent SDQ-25 total score differences and patient characteristics

The relation between patient-parent SDQ-25 total score differences and patient characteristics underwent analysis. For example, we investigated if a younger age, longer disease course, different disease course or higher active joint count led to disparities between parent and patient perceptions of mental health and applied Spearman’s correlations of patients' age, disease course, disease duration, or active joint count with patient—parent total score differences. It emerged that weight was related to differences in perception of peer relation problems (rs = -0.43), disease course and differences in perception of conduct problems (p = 0.045) and pro-social behavior (p = 0.002) were connected, and active joint count was associated with differences in perception of hyperactivity/inattention (rs = 0.44) (for details see Table 8).

**Table 8:** Correlation between SDQ-25 parent-patient differences and patient characteristics.

SDQ-25: parent-patient differences	Gender	Age	Weight	Height	Disease course	Disease duration	Active joint count
Emotional symptoms	p = 0.136	rs = 0.34	rs = 0.01	rs = -0.01	p = 0.020*	rs = 0.06	rs = 0.26
Conduct problems	p = 0.459	rs = 0.02	rs = 0.06	rs = -0.12	p = 0.045*	rs = 0.24	rs = -0.31
Hyperactivity/inattention	p = 0.560	rs = -0.02	rs = 0.24	rs = 0.04	p = 0.454	rs = -0.17	rs = 0.44*
Peer relation problems	p = 0.428	rs = -0.31	rs = -0.43*	rs = -0.17	p = 0.618	rs = 0.02	rs = -0.15
Pro-social behavior	p = 0.958	rs = 0.07	rs = -0.25	rs = -0.28	p = 0.002*	rs = 0.26	rs = 0.26
Total score	p = 0.185	rs = -0.03	rs = -0.07	rs = -0.16	p = 0.508	rs = 0.10	rs = 0.04

* p < 0.05

A summary of the associations between parent-patient differences in PODCI standardized scores and sample characteristics is presented in Table 9.

**Table 9:** Correlation between PODCI Adolescent Outcomes Questionnaire parent-patient differences and patient characteristics.

PODCI Adolescent Outcomes Questionnaire: parent- patient differences	Gender	Age	Weight	Height	Disease course	Disease duration	Active joint count
Upper Extremity and Physical Function Scale	p = 0.762	rs = -0.03	rs = 0.21	rs = 0.25	p = 0.628	rs = -0.17	rs = -0.05
Transfer and Basic Mobility Scale	p = 0.307	rs = 0.41*	rs = 0.11	rs = 0.36	p = 0.307	rs = -0.06	rs = 0.06
Sports/Physical Functioning Scale	p = 0.486	rs = 0.02	rs = 0.10	rs = 0.26	p = 0.857	rs = -0.10	rs = 0.15
Pain/Comfort Scale	p = 0.702	rs = -0.17	rs = 0.16	rs = -0.03	p = 0.702	rs = 0.09	rs = 0.41*
Happiness Scale	p = 0.421	rs = -0.13	rs = 0.27	rs = 0.22	p = 0.736	rs = -0.37	rs = 0.40*
GLOBAL FUNCTIONING SCALE	p = 0.368	rs = 0.04	rs = 0.25	rs = 0.28	p = 0.520	rs = -0.03	rs = -0.05

* p < 0.05

The data acquired, as evaluated by associations with the active joint count, reveals moderate significant correlations in the Pain/Comfort and Happiness Scales (rs = -0.41 and 0.40). A higher active joint count was connected to bigger differences in the perception of pain/comfort and happiness between parents and their children.

Patients' age was related to the difference in parent-patient reports in the Transfer and Basic Mobility Scale (rs = 0.41). The older the patient, the bigger difference in the perception of ability to transfer and basic mobility, between parents and their children.

### Cross-group comparisons between the PODCI and the SDQ-25 for patient and parent results

As indicated in Table 10, the study groups do not differ significantly in regards to either the PODCI or SDQ-25 results, with reference to the general and individual subscales results.

**Table 10:** Results of comparisons between the PODCI and SDQ-25: Patient and parent results.

PODCI Adolescent Outcomes Questionnaire	Comparison between parent and patient results	SDQ-25	Comparison between parent and patient
Upper Extremity and Physical Function Scale	p = 0.372	Emotional symptoms	p = 0.627
Transfer and Basic Mobility Scale	p = 0.536	Conduct problems	p = 0.960
Sports/Physical Functioning Scale	p = 0.805	Hyperactivity/inattention	p = 0.425
Pain/Comfort Scale	p = 0.985	Peer relation problems	p = 0.483
Happiness Scale	p = 0.898	Pro-social behavior	p = 0.707
GLOBAL FUNCTIONING SCALE	p = 0.742	Total score	p = 0.500

## Discussion

The study focused on the comparison of assessments of perceived mental health and disease experience in a group of adolescents with JIA and their parents. The reports from parents and patients were acquired using valid and reliable measures. As 100% of the study group completed the test-retest study, it was not necessary to analyze the refusal rate. Demographic and disease-related data on the participants was collected.

The psychometric properties of the Polish version of the PODCI (parent- and self- report for adolescents aged 11–18 years) may support the interpretation of scores enabling decisions concerning individual patients. The reliability of the original version of the PODCI, where Cronbach's alpha ranged from 0.76 (Happiness Scale) to 0.95 (Transfers And Mobility Scale) [14], is consistent with our results, in which Cronbach's alpha peaked at 0.88 and 0.89 for the parent and adolescent Global Functioning Scales, respectively, measured from 0.63 (Upper Extremity and Physical Function Scale for the parent version) and 0.76 (Basic Mobility Scale for the patient report) to 0.90 (Sports/Physical Functioning Scale for the patient version) and 0.92 (Sports/Physical Functioning Scale for the parent version) indicating, in general, high internal consistency.

The test—retest agreement of the original, English-language version of the PODCI revealed Pearson correlations which ranged from 0.71 (Happiness and Satisfaction Scale) to 0.97 (Transfers and Mobility Scale) for a subset of parents who completed the same questionnaire a second time after 1 to 2 days [18]. Our study achieved a higher test—retest reliability ranging from 0.80 (Transfers And Mobility Scale) to 0.99 (Upper Extremity and Physical Function Scale) in the parent report and from 0.94 (Happiness Scale) to 1.0 (Sports/Physical Functioning and Upper Extremity and Physical Function Scales) in the patient report. Our results confirmed concurrent validity of the PODCI only for the parent version. No statistically significant correlations between Patient PODCI and SDQ-25 results were revealed.

Research carried out by Ding et al. [30] confirmed that there were no significant differences in the level of psychological difficulties in children and adolescents with polyarthritis face compared to children and adolescents in the normal population. Ding et al. also revealed that the psychological functioning of children and adolescents with polyarthritis is related to physical disability but not with disease activity. In a study by LeBovidge et al. [31], adolescents with arthritis were not found to be at greater risk of poor self-concept than the controls, although the authors highlighted the fact that there were few references to this issue in studies. The type of control group involved may well impact results so researchers need to exercise caution regarding their conclusions about the self-concept of children with arthritis when referring only to comparisons with normative data. In such situations there is a danger that the extent to which arthritis affects self-esteem may be underestimated [31].

In their review of published studies, Rapoff et al. [32] concluded that there is no evidence of significant psychosocial deficits in children with JIA compared with normative or healthy control samples. Bearing these inconsistencies in mind, our study showed a significant decrease in the PODCI Sports/Physical Functioning Scale, Pain/Comfort Scale, Happiness Scale and the Global Functioning Scale, when compared with normative samples. However, in the case of the SDQ-25, the overall result compiled from all the domains and the general results was “psychiatric disorder: unlikely” in both the patient and parent versions.

Good levels of agreement have been previously reported between parents and children in rating JIA dysfunction [16]. Parents were accurate in reporting the level of their child's physical functioning as measured by the Juvenile Arthritis Functional Assessment Report for Children and Parents questionnaires (JAFAR-C and JAFAR-P) as demonstrated by the Baildam et al. study [33], although there was no parental measure of expectation or perceived competence.

Ernett et al. [34] performed a study that was similar to the presented study, the results of which revealed that a child's and their mother's reports of the child's perceived competence are not comparable, nor are their perceptions of how JIA is experienced by the child. In two of the four domains of functioning (athletic competence and acceptance by peers) mothers gave a lower rating of their child's competence than did the child themselves. The mothers also perceived the disease as having a more substantial impact on family life than did their children [34].

In their 1998 study, Billings et al. [35] revealed agreement between parent-reports and the reports of children (aged 10 years and older) regarding the pain and disability associated with arthritis. They also uncovered differences in reporting on the psychological impact in the comparison of parent- and self-report. Parents admitted to the existence of more mood and psychological symptoms than did their children. This may be due to biases in the parent reports, it is also possible that some children may be less aware of the difficulties they experience than their parents.

Sawyer et al. [36] revealed that compared to the children, parents reported significantly lower scores (worse HRQL) in five of the eight PedsQLTM scales used to rate children's HRQL. In our study, the results did not reveal any discrepancies between patients' and parents' perceptions of mental health (SDQ-25) and overall health, pain and ability to participate in normal daily activities, as well as more vigorous activities typically associated with young people in the (PODCI evaluation). This suggests that our assumption about patient-parent discrepancies could not be confirmed and that parents and adolescents share similar perceptions of the patient's health and a good mutual understanding exists between parents and adolescents with JIA.

In a study by Baildam et al. [33] no clear link between the severity of physical disease and psychological function was found. Despite incidence of increased behavioral symptoms in children with arthritis, Baildam et al. [33] support the view that there is no clear link between disease severity and psychological symptomatology. They highlighted the current trend to place more value on the coping resources and strategies of families and the complexity of parental and sibling relationships [33]. It may be that all of these play a greater role in determining psychological outcome than the disease itself.

Vandvik and Eckblad's study [37] presents the viewpoint that any psychological dysfunction may be connected to factors other than the degree of illness in itself. Mothers of children with JIA often exhibited trait anxiety and this is rather due to psychosocial background variables such as their maternal childhood environment and chronic family difficulties, than to the disease variables.

A formal assessment of family stress in the present study was not feasible, but the degree of positive correlation shown between parental and child scores indicates that this specific patient group has good mutual understanding. Communication within the family appears healthy and there is a lack of emotionally based distortions.

The patients displayed relatively satisfactory psychiatric adjustment, probably a reflection of their family's positive coping strategies. The JAFAR questionnaire has proven to be a useful study instrument. Levels of self-esteem and depression in the study group did not differ to any significant extent from the healthy population. Behavioral symptoms were higher than in healthy controls although no correlation with any of the physical parameters was registered [33].

The focus of our study was on the relationships between psychological dysfunction and the degree of illness, specifically in the context of differences between the patient and parent results. Associations between parent/patient differences in PODCI standardized scores and sample characteristics revealed that a higher active joint count was related to greater disparity in the perception of pain/comfort and happiness. There was a bigger difference in the perception of ability to transfer and basic mobility, between parents and their children as the age of the patient increased. In the SDQ-25 results, disease course was linked to differences in perception of conduct problems and pro-social behavior, active joint count was associated with differences in perception of hyperactivity/inattention. Overall, differences between parental and adolescent perspectives become greater as disease severity (disease course, active joint count) and the age of the patients increases.

## Study limitations and future research implications

The strength of our study lies in the use of reports from both parents and children obtained using valid and reliable measures of physical and psychological function in a homogeneous group of adolescents with JIA and their parents. The fact that all the children included in this study were recruited from the same rheumatology clinic is considered a limitation of the study.

We therefore advise caution if generalizing the results of this study to children receiving treatment in other settings. The correlation and cross-sectional design of this study preclude the drawing of firm causal inferences from these results, in particular, the important relationship between pain/comfort and inattention, and active joint count. This issue may be addressed in longitudinal data analyses. Further evaluation of the criterion validity of the patient version in respect of the psychometric properties of the PODCI Polish-language version is needed. In order to broaden the generalizability of our study in future research, a larger and more representative sample would be required, and we suggest the collection of longitudinal data to enable better evaluation of the directional effects of causal relations.

## Conclusions

Careful consideration is required before conclusions are made concerning psychosocial deficits in children with JIA in comparison to normative samples. As this study reveals, particular areas exhibit a significant decrease although this drop may depend on the applied study measure. In addition, very similar perceptions of patients’ health were reported by parents and adolescents with JIA. It is important to emphasize that differences in this perspective seem to widen as the severity of the disease (disease course, active joint count) and the age of the patients increases. Despite the differences between the perspectives of parents and children, each individual participant is a potentially valuable source of clinical information on the level of psychological adjustment to chronic disease in adolescents.

The translation and adaptation process of the PODCI questionnaire delivers an analogous instrument to be used in the assessment of adolescents with JIA in terms of overall health, pain, and ability to participate in activities. The excellent internal consistency, intrarater and test-retest reliability of the Global Functioning Scale means it may prove extremely useful in attempts to identify patients reporting significant problems in this potentially high risk group.

## Supporting information

S1 File Patient data and SDQ-25. (XLS)

S2 File PODCI AdolescentScoring_ParentReport-1st completion. (XLS)

S3 File PODCI AdolescentScoring_SelfReport_1st completion. (XLS)
