# Correction: Mental health and adjustment to juvenile idiopathic arthritis: Level of agreement between parent and adolescent reports according to Strengths and Difficulties Questionnaire and Adolescent Outcomes Questionnaire

**DOI:** 10.1371/journal.pone.0176794

**Published:** 2017-04-26

**Authors:** The PLOS ONE Staff

## Notice of Republication

This article was republished on March 14, 2017, as a Supporting Information file was published in error. The publisher apologizes for the error. Please download this article again to view the correct version.
